# First ever programmatic implementation of triple drug therapy in the prevention of lymphatic filariasis in Haiti: lessons learned in improving the quality and coverage of locally led programming in lymphatic filariasis-persistent and conflict-affected endemic communes

**DOI:** 10.1093/inthealth/ihag042

**Published:** 2026-05-08

**Authors:** Katina Sommers, Wedsanley Jean Philippe, Gianni C Decastro, Merilien Jean-Baptiste, Farah-Nelhy Momprévil

**Affiliations:** IMA World Health, 1730 M St NW #1100, Washington, DC 20036, USA; IMA World Health, Port-au-Prince, HT6140, Haiti; IMA World Health, Port-au-Prince, HT6140, Haiti; Coordination Nationale des Programmes Nationaux de Malaria et de Filariose Lymphatique, Haiti Ministry of Public Health and Population, Port-au-Prince, HT6110, Haiti; Coordination Nationale des Programmes Nationaux de Malaria et de Filariose Lymphatique, Haiti Ministry of Public Health and Population, Port-au-Prince, HT6110, Haiti

**Keywords:** elephantiasis, filarial, feasibility studies, global health, Haiti, mass drug administration, public health

## Abstract

Although significant strides have been made towards lymphatic filariasis (LF) elimination in Haiti, which has experienced ongoing multidimensional crises, 19 out of 140 communes still require mass drug administration (MDA) despite multiple rounds using the two-drug regimen of diethylcarbamazine (DEC) and albendazole. This includes the commune of Limonade, which has experienced persistently high LF prevalence despite nine rounds of two-drug MDAs since 2010. In September and October 2023 and July 2024, the Haitian Ministry of Public Health and Population, with support from IMA World Health and the US Centers for Disease Control and Prevention, adapted the standard WHO strategy, proving the feasibility, acceptability and scalability of LF MDA with three drugs (ivermectin, DEC and albendazole [IDA]) through the successful implementation of two rounds of IDA MDA in Limonade.

## Introduction

Haiti, one of four countries in the Americas that remain endemic for lymphatic filariasis (LF),^1^ a debilitating neglected tropical disease, has faced multidimensional crises with compounding shocks, including economic turmoil, political upheaval, near collapse of the healthcare and education systems, disease outbreaks, environmental risks and unprecedented levels of insecurity. Following the July 2021 assassination of President Jovenel Moïse, violence has steadily increased, with gangs controlling the majority of the capital, Port-au-Prince. In 2025, an estimated 4.2 million people (~32% of the population) are expected to require health assistance in Haiti,^2^ with >5 million people facing food insecurity. Access to safe and improved water, sanitation and hygiene is limited, with 35% of Haitians lacking access to safe drinking water.^3^ The widespread presence of armed groups, which have blockaded all national road routes, has hindered access across the country, leading to extended fuel shortages and increased operations costs for humanitarian and civilian actors.

In light of this challenging operational context, Haiti has made significant strides towards the elimination of LF, a mosquito-borne disease that can lead to lymphoedema (elephantiasis) and scrotal swelling (hydrocele), which may result in permanent disability, stigma and poverty due to the social, economic and mental impacts of the disease.^4^ In 2023, 657 million people, across 39 countries, lived in areas at risk of LF.^1^ As of 2023, ~30% (4 487 915 people) of the population in Haiti required LF mass drug administration (MDA).^1^ To interrupt transmission, the WHO recommends preventive chemotherapy through annual MDA for the entire at-risk population, with a minimum threshold of 65% coverage.^1^ Several clinical trials, including in northern Haiti, have shown that MDA with the three-drug regimen of ivermectin, diethylcarbamazine and albendazole (IDA) is more effective than the two-drug regimen and can be a useful tool for LF elimination.^5^

As of April 2025, an impressive 121 out of 140 communes in Haiti have stopped treatment for LF and entered a post-MDA surveillance phase of the LF programme. In alignment with the Ministry of Public Health and Population (MSPP) strategic plan to eliminate LF, IDA MDA was implemented for the first time programmatically in September and October 2023 in the commune of Limonade, with the aim of proving the feasibility, acceptability and scalability of IDA MDA in the LF-endemic communes in Haiti. Limonade, a commune in the relatively stable North Department, has undergone nine rounds of two-drug MDA since 2010, although LF remains endemic.

This case study showcases the success of the two rounds of IDA MDA that took place in 2023 and 2024 in Limonade, led by the MSPP, with support from IMA World Health (IMA) and the US Centers for Disease Control and Prevention (CDC). Contextual challenges were overcome through strong coordination, an extended microplanning period with intensive community engagement to foster improved local ownership and acceptance, as well as adapting implementation to areas of relative calm, where access was feasible. This is the first time an IDA MDA has been rolled out programmatically in Haiti, and the high reported coverage (2023: 74.4%; 2024: 68.0%) serves as a foundation for scaling up IDA MDA in the remaining endemic communes. The findings, successes, challenges and recommendations can be applicable to other contexts in the scaling up of new MDA modalities and in fragile, conflict and/or resource-limited settings.

## Planning

Prior to IDA MDA implementation, stakeholder engagement activities took place to ensure maximum coverage. Key preimplementation activities included (Table 1):

- **Strategic planning**: In the initial project period, strategic planning discussions across the Haiti LF landscape took place, including strong communication and collaboration among the MSPP (at all levels), LF implementing partners, communities, IMA and CDC. Frequent virtual and in-person coordination meetings were held. Contingency plans were drafted, in the event the MDA modality or timing required revisions due to issues of insecurity and limited access.
- **School census:** School-based distribution posed a challenge due to the presence of unregistered schools. To mitigate this, the MSPP conducted a school census in 2022 (using Ministry of Education data) to determine the number of functional schools, ensuring that no students were missed.
- **Microplanning:** Microplanning was a crucial element in identifying key stakeholders, assessing resource requirements and availability, recruitment, and determining distribution sites and modalities, including alternative modalities, should the MDA need to be adapted for the evolving security context. Active engagement with local authorities, religious leaders and community members was vital in facilitating comprehensive planning and implementation. A three-pronged distribution strategy was established, including fixed-point, door-to-door and school-based distribution.
- **Information, education, and communication (IEC) and training materials**: Tools were refined and updated to include changes resulting from the transition from two- to three-drug MDA and to address the concerns of never-treated individuals.
- **Recruitment and training**: High performing staff from previous MDAs were recruited. Where possible, staff were hired from the local areas. In 2023, a total of 233 personnel were trained, and in 2024, 195 staff were trained. Cadres included community sensitizers, community leaders, village and street criers for awareness messaging, data recorders, community drug distributors, supervisors and clinical healthcare workers trained in adverse and severe adverse event management.
- **Social mobilisation**: Social mobilisation and community outreach took place through the training and deployment of criers and community leaders, the use of a sound truck and recruiting social media influencers to spread awareness about the IDA MDA campaign. Building on the learnings from the first round, an extended 2024 social mobilisation period took place over 2 months.

**Table 1:** Key MDA planning activities.

Activity	Objective	Activities	Outcome
School census (2022)	Improve school information and provide detailed data on the population of school-aged children	-Meeting school officials -Training enumerators -12-day census	67/125 active schools identified (14 513 students) from Ministry of Education school list
Microplanning (2023, 2024)	Ensure key actors play an active role in informing IDA MDA planning and implementation, as context-specific information is vital in achieving maximum coverage and providing accessible and equitable services in hard-to-reach areas	-Meeting key stakeholders (departmental/commune staff, local leaders, school administrators, religious leaders, community groups)	-Determination of number of teams, locations for fixed posts, detailed implementation and distribution plan -Establishment of technical committee
Review and revision of IEC and training materials (2023, 2024)	Collaborate and harmonize among LF partners and the MSPP in the drafting and finalizing of IDA IEC and training materials	-2-day workshop	-Development of training curriculum -Updated IEC materials
Recruitment/training (2023, 2024)	Recruit and train high calibre staff (across all cadres) to implement the MDA, equipping them with the skills to manage adverse events, lead community engagement and awareness efforts and implement an MDA with a minimum of 65% coverage	-Training clinical staff on adverse and severe adverse event management -Training all distribution team cadres on awareness messaging, materials and drug management, distribution strategy and data-collection techniques	-Staff trained at three clinics on adverse event management -Recruitment and training of MDA distribution teams (2023: 58, 2024: 50) with one drug distributor, one data recorder and one crier (mobilizer) per team
Social mobilisation (2023, 2024)	Train and deploy trusted community members to generate awareness and mobilize the community in advance of the MDA	-Deployment of community leaders and mobilizers, sound truck, social media engagement, radio talk shows	-High level of community awareness and acceptance of IDA MDA, leading to ≥65% coverage

## Implementation

Following social mobilisation, the first round of IDA MDA was rolled out in September 2023. A second round of IDA MDA took place in July 2024. Both rounds were 10 days in length, with an additional 4 days of mop-up in targeted areas of low coverage, identified during supervision using the supervisor’s coverage tool (SCT). Distribution took place at fixed posts and through door-to-door and school-based modalities. Participants were treated under direct observation, with pills administered and ingested in the presence of a community drug distributor. In 2024, schools were not a primary focus, as they were not in session due to ongoing national insecurity. To maximize coverage in light of this challenge, community events such as market days, voodoo and church ceremonies and sporting events were targeted. Based on community feedback, evening distribution was implemented (where security allowed) so that community members could take the medication after work hours and before bed.

Data were collected electronically, enabling real-time tracking of coverage during the MDA. First-level supervisors implemented the SCT midway through the distribution to identify areas at <65% coverage threshold. This allowed distribution teams to adjust their strategies and concentrate their efforts on treatment and mop-up activities in localities with low coverage. In 2023, 74.4% of the total at-risk population (45 265 participants/60 815 population) in Limonade was reached, and in 2024, 68.0% (42 378 participants/62 319 population) of the at-risk population was treated. Refusals across both rounds of MDA were low (2023: 0.7%, 2024: 1.0%), with primary concerns related to the fear of side effects and a preference for taking medication at home.

Three health facilities were designated to manage adverse and severe adverse events, although these were minimal (2023: 0.1%, 2024: 0.1%).

## Conclusion

One of the primary drivers of the successful implementation of IDA MDA in Limonade was detailed microplanning and intensive social mobilisation, supported by strong local engagement and collaboration. As a persistent LF commune, there was initial uncertainty about how residents would respond to the introduction of a third MDA drug; however, despite significant logistical challenges, including fuel shortages in 2023, delays in MDA supply delivery due to road blockades requiring air transport, as well as high implementation costs in Haiti, the pilot was successfully executed through strong community engagement, awareness and acceptance. Stakeholders across all levels of the health system (national, departmental and communal) were engaged from the outset to ensure coordination and application of lessons learned, while the MSPP led MDA staff training, building capacity that supported subsequent scaling up to eight additional endemic communes. Although WHO-recommended epidemiological monitoring surveys have not been conducted due to funding constraints, limiting the assessment of impact on LF prevalence, the high coverage achieved demonstrates the feasibility, acceptability and scalability of rolling out IDA MDA in the remaining endemic communes in Haiti, moving the needle closer to the target of national LF elimination. Areas facing complex humanitarian emergencies, such as Haiti, must utilize the most effective tools available, such as IDA, to combat LF and enhance health outcomes for their populations in the shortest time possible.

## Data Availability

The data underlying this article will be shared on reasonable request to the corresponding author.

## Notes

Note: The funding language used is required verbatim from CDC.
